# Magnesium nebulization utilization in management of pediatric asthma (MagNUM PA) trial: study protocol for a randomized controlled trial

**DOI:** 10.1186/s13063-015-1151-x

**Published:** 2016-05-24

**Authors:** Suzanne Schuh, Judy Sweeney, Stephen B. Freedman, Allan L. Coates, David W. Johnson, Graham Thompson, Jocelyn Gravel, Francine M. Ducharme, Roger Zemek, Amy C. Plint, Darcy Beer, Terry Klassen, Sarah Curtis, Karen Black, Darcy Nicksy, Andrew R. Willan, on behalf of Pediatric Emergency Research Canada Group

**Affiliations:** Division of Paediatric Emergency Medicine, The Hospital for Sick Children, Child Health Evaluative Sciences, SickKids Research Institute, University of Toronto, 555 University Avenue, Toronto, ON M5G 1X8 Canada; Sections of Pediatric Emergency Medicine and Gastroenterology, Alberta Children’s Hospital, Alberta Children’s Hospital Research Institute, University of Calgary, 2888 Shaganappi Trail NW, Calgary, AB T3B 6AB Canada; Departments of Paediatrics, Pharmacology and Physiology, Alberta Children’s Hospital Research Institute, Faculty of Medicine, University of Calgary, C4,643, 2888 Shaganappi Trail NW, Calgary, AB T3B 6AB Canada; Division of Pediatric Emergency Medicine, Alberta Children’s Hospital, University of Calgary, 2888 Shaganappi Trail NW, Calgary, AB T3B 6AB Canada; Division of Paediatric Emergency Medicine, Department of Pediatrics, Centre Hospitalier Universitaire Ste-Justine, Université de Montréal, 3175 Cote Sainte-Catherine, Montreal, QC H3T 1C5 Canada; Department of Pediatrics, Centre Hospitalier Universitaire Ste-Justine, Université de Montréal, 175 Cote Sainte-Catherine, Montreal, QC H3T 1C5 Canada; Division of Pediatric Emergency Medicine, Children’s Hospital of Eastern Ontario (CHEO), 401 Smyth Road, Ottawa, ON K1H 8L1 Canada; Division of Emergency Medicine, Children’s Hospital of Eastern Ontario (CHEO), 401 Smyth Road, Ottawa, ON K1H 8L1 Canada; Divsion of Pediatric Emergency Medicine, The Children’s Hospital of Winnipeg, University of Manitoba, 820 Sherbrook Street, Winnipeg, MB R3J 1R9 Canada; Children’s Hospital Research Institute of Manitoba (formerly Manitoba Institute of Child Health), Academic Faculty of Medicine, 715 McDermot Ave, Winnipeg, MB R3E 3P4 Canada; Department of Pediatrics and Child Health, University of Manitoba, 715 McDermot Ave, Winnipeg, MB R3E 3P4 Canada; Child Health Program, Winnipeg Health Region MICH, 715 McDermot Ave, Winnipeg, MB R3E 3P4 Canada; Division of Paediatric Emergency Medicine, Stollery Children’s Hospital, University of Alberta, 8440 112 Street Northwest, Edmonton, AB T6G 2B7 Canada; Division of Pediatric Emergency Medicine, University of British Columbia, BC Children’s Hospital, 4480 Oak St, Vancouver, BC V6H 3N1 Canada; SickKids Research Institute, The Hospital for Sick Children, University of Toronto, 555 University Avenue, Toronto, ON M5G 1X8 Canada; Child Health Evaluative Sciences, SickKids Research Institute, Dalla Lana School of Public Health, University of Toronto, 555 University Avenue, Toronto, ON M5G 1X8 Canada; SickKids Research Institute, The Hospital for Sick Children, 555 University Ave, Toronto, ON M5G 1X8 Canada

**Keywords:** Randomized controlled trial, children, acute asthma, magnesium

## Abstract

**Background:**

Up to 30 % of children with acute asthma are refractory to initial therapy, and 84 % of this subpopulation needs hospitalization. Finding safe, noninvasive, and effective strategies to treat this high-risk group would substantially decrease hospitalizations, healthcare costs, and the psycho-social burden of the disease.

Whereas intravenous magnesium (Mg) is effective in severe refractory asthma, its use is sporadic due to safety concerns, with the main treatment goal being to prevent intensive care unit admission. In contrast, nebulized Mg is noninvasive, allows higher pulmonary drug concentrations, and has a much higher safety potential due to the lower rate of systemic delivery. Previous studies of inhaled Mg show disparate results due to the use of unknown/inefficient delivery methods and other methodological flaws.

**Methods/Design:**

The study is a randomized double-blind controlled trial in seven Canadian pediatric Emergency Departments (two-center pilot 2011 to 2014, Canada-wide November 2014 to December 2017). The trial will include 816 otherwise healthy children who are 2 to 17 years old, having had at least one previous wheezing episode, have received systemic corticosteroids, and have a Pediatric Respiratory Assessment Measure (PRAM) ≥ 5 points after three salbutamol and ipratropium treatments for a current acute asthma exacerbation. Eligible consenting children will receive three experimental treatments of nebulized salbutamol with either 600 mg of Mg sulfate or placebo 20 min apart, using an Aeroneb Go nebulizer, which has been shown to maximize pulmonary delivery while maintaining safety. The primary outcome is hospitalization within 24 h of the start of the experimental therapy for persistent respiratory distress or supplemental oxygen. Secondary outcomes include all-cause hospitalization within 24 h, PRAM, vital signs, number of bronchodilator treatments by 240 min, and the association between the difference in the primary outcome between the groups, age, gender, baseline PRAM, atopy, and “viral induced wheeze” phenotype (Fig. 1).

**Discussion:**

If effective, inhaled Mg may represent an effective strategy to minimize morbidity in pediatric refractory acute asthma. Unlike previous works, this trial targets nonresponders to optimized initial therapy who are the most likely to benefit from inhaled Mg. Future dissemination of results will include knowledge translation, incorporation into a Cochrane Review, presentation at scientific meetings, and a peer-reviewed publication.

**Trial registration:**

NCTO1429415, registered 2 September 2011.

**Electronic supplementary material:**

The online version of this article (doi:10.1186/s13063-015-1151-x) contains supplementary material, which is available to authorized users.

## Background

Acute asthma is a leading cause of pediatric emergency department (ED) visits and of pediatric hospitalizations [1], with the annual asthma expenditures in the United States exceeding $37 billion [2]. In 2005, 754,000 pediatric ED asthma visits were reported in the US [3, 4], and 27 % of these resulted in hospitalization [4]. Practice guidelines recommend high dose ß2 agonists, inhaled anticholinergics, and oral corticosteroids to manage major acute exacerbations [5–9]. However, this initial optimal therapy [9–11] is not always effective [12] because up to 30 % of patients with asthma are resistant to initial bronchodilation [13] in part due to ß2 adrenoreceptor gene polymorphism [14–32], and gene polymorphisms also impact the frequently delayed response to corticosteroids [33–35]. Finding further effective strategies to decrease pediatric asthma hospitalizations in this high-risk population is necessary.

Magnesium sulfate (Mg) produces rapid bronchodilation by multiple mechanisms [36–39] and can be given either intravenously (IV) or by nebulization. Two key meta-analyses of adult and pediatric trials have confirmed that the addition of IV Mg to routine therapy significantly decreases hospitalizations and improves lung function [40, 41]. These authors, as well as various asthma guidelines, recommend that IV Mg be considered in children not responding to initial management [40, 42–44]. However, IV Mg is used infrequently and often as a last resort [45, 46]. In contrast, the nebulization route is noninvasive and offers a major advantage of targeted delivery to the lower airway and less potential for side effects [47]. However, the benefit of nebulized Mg has not been well established.

The investigation of the efficacy of nebulized Mg has been limited, with disparate results. The dose of Mg varied seven-fold among studies [48–60], and only one study [48] limited participants to nonresponders to bronchodilators. Two meta-analyses of mainly adult studies have found a trend toward reduction in hospitalizations in patients given nebulized Mg [40, 61], with the recent Cochrane review reaching similar conclusions [59]. The main limitations of past pediatric studies are failure to limit participants to nonresponders to bronchodilators, inadequate use of anticholinergics, use of inefficient delivery methods, and small sample size [49, 58, 60, 62]. In contrast, this study focuses on the poor responders to optimized baseline acute asthma therapy. This group is at the highest risk of hospitalization and the most likely to benefit from nebulized Mg intervention. Furthermore, previous studies have used nebulizers of suboptimal/unknown efficacy. In contrast, the device used in this study has been pre-tested and shown to deliver a considerably greater proportion of the drug into the lungs compared to the conventional nebulizers.

### Research questions and study hypothesis

#### Primary question

The primary question is whether, in children 2–17 years of age with acute asthma who have persistent moderate to severe airway obstruction despite maximized initial bronchodilator and steroid therapy, a reduction can be achieved in the probability of hospitalization within 24 h of starting experimental therapy. Specifically, this trial compares those patients who receive three nebulized Mg and salbutamol treatments to those receiving only nebulized salbutamol.

#### Secondary questions

The trial seeks to determine the following for these treatment modalities:

- Is there a difference in the probability of all-cause hospitalization within 24 h of starting therapy?
- Is there a difference in the changes in the validated Pediatric Respiratory Assessment Measure (PRAM), respiratory rate, oxygen saturation, and blood pressure to 240 min?
- Does the treatment effect with respect to primary outcome vary among the subgroups defined by the following variables: age, sex, pre-randomization PRAM score, personal history of atopy and “viral-induced wheeze” phenotype?

#### Hypothesis

We hypothesize that the children with PRAM ≥5 points after optimized initial inhaled bronchodilator and systemic steroid therapy who are given nebulized Mg in addition to nebulized salbutamol will have a significantly lower probability of hospitalization compared to those given salbutamol only.

## Methods/Design

### Study design and population

This is a seven-center, randomized, double-blind controlled trial. Two study groups are compared: nebulized salbutamol with Mg sulfate and nebulized salbutamol with placebo. This study has received approval by the Research Ethics Board of the Hospital for Sick Children. Furthermore, local Research Ethics Boards have approved the study at all participating sites (see Additional file 1). A written informed consent and assent where appropriate is being obtained from all study participants.

All otherwise healthy children 2–17 years of age with a past history of asthma/recurrent wheeze arriving in the ED when the study nurses are on duty with a presenting PRAM ≥5 are considered potentially eligible. Following assessment by the ED physician, the patients receive oral/IV corticosteroid plus three salbutamol and ipratropium inhalations according to the local management guidelines [63–68]. If the PRAM is still ≥5 after the initial therapy, the study nurse confirms eligibility, measures the pre-randomization PRAM score, and obtains informed consent.

#### PRAM score

PRAM is a validated 12-point clinical asthma severity score [69], exhibits the most comprehensive measurement properties of all asthma scores [70], and has been successfully used as an outcome in major trials [71]. It is the only score with demonstrated criterion validity, using respiratory resistance as the gold standard [72]. PRAM has been validated in both preschool and school-aged children who have been assessed in the ED for asthma and has a strong association with admission [73]. The score has inter-rater reliability consistently above 70 % [73] and is currently implemented in virtually all pediatric EDs across Canada. Children with PRAM ≥5 following initial bronchodilator therapy have at least a 30 % probability of hospitalization and represent 84 % of all acute asthma admissions in children. All participating EDs now measure the PRAM score as part of routine clinical assessment in their EDs in children with acute asthma. Because Calgary is situated 1000 m above sea level, oxygen saturations there can be expected to be approximately 2 % lower than in Toronto (International Civil Aviation Organization, Manual of the ICAO Standard Atmosphere, Doc 7488-CD, Third Edition, 1993, ISBN 92-9194-004-6). Therefore, the oxygen saturation component of the PRAM is adjusted in Calgary.

#### Inclusion criteria

Inclusion criteria are (1) 2 to 17 years of age, (2) diagnosis of asthma/reactive airways/viral-induced wheeze plus at least one prior acute episode of wheezing treated with inhaled ß2 agonists or oral corticosteroids, (3) persistent moderate to severe airway obstruction after oral steroid and three doses of salbutamol and ipratropium, defined as a PRAM ≥5 [73].

#### Exclusion criteria

Exclusion criteria are (1) no past history of wheezing or bronchodilator therapy; (2) prior IV Mg therapy during the index visit; (3) critically ill children requiring immediate intubation; (4) coexistent renal, chronic pulmonary, neurologic, cardiac, or systemic disease; (5) children who in the opinion of the treating physician require a chest radiograph due to atypical clinical presentation and are diagnosed to have lobar consolidation with pneumonia, felt to be the primary cause of respiratory distress; (6) known hypersensitivity to Mg sulfate; (7) patients previously enrolled; (8) insufficient command of the English or French language; and (9) lack of a phone.

#### Delivery device

To ensure adequate delivery of the experimental treatment into the lungs, we have pre-tested the Aeroneb™ Go Micropump Nebulizer along with the Idehaler™ Pocket system without valves that connects with a facemask in vitro and in vivo. We found that this device deposited 20 % of the charge dose compared to 4 % by conventional nebulizers, while also maintaining acceptable osmolarity and safety [74]. This device has been distributed to all participating sites, related training provided, and Health Canada approval obtained. All experimental treatments are delivered with this device. This device is fully licensed both in the United States and in Europe and is currently awaiting license approval in Canada. We do not anticipate any difficulties with its official adoption by the time this trial is concluded.

### Sample selection

Children presenting to the collaborating EDs at The Hospital for Sick Children, Children’s Hospital of Eastern Ontario, Ste Justine’s Hospital, Alberta Children’s Hospital, Stollery Hospital, Children’s Hospital of Winnipeg and the B.C. Children’s Hospital who meet eligibility criteria are approached for enrollment when the research nurses are on duty. The research nurses keep a log of all children presenting to the ED with acute asthma during the study period whether randomized or not in order to assess the generalizability of the study. All aforementioned hospitals are tertiary care centers, which see the entire clinical and demographic spectrum of the asthma population.

### Pre-study screening and baseline evaluation

All previously healthy children 2 and 17 years of age with acute asthma have a PRAM score measured in triage. Those meeting local ED criteria for enhanced therapy receive either oral dexamethasone, oral prednisolone/prednisone, or IV hydrocortisone plus three salbutamol and ipratropium inhalations via a Metered Dose Inhaler/Valved Holding Chamber (MDI/VHC)/nebulizer according to the local asthma pathway and 20 min apart.

### Study procedures

**Fig. 1:**
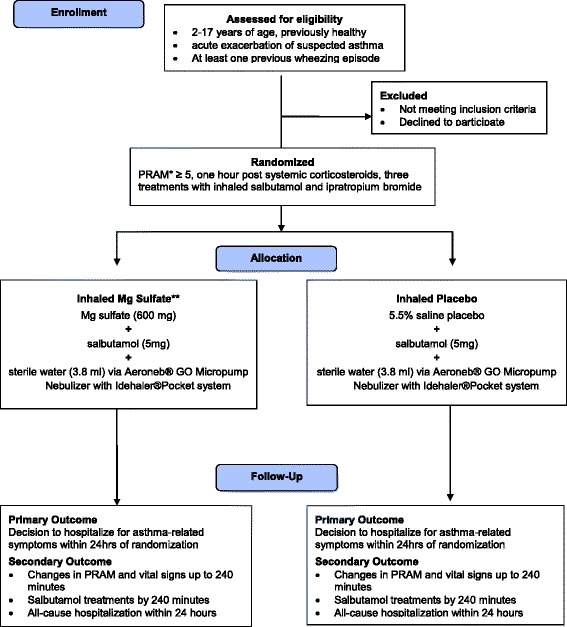
Flow diagram of participant study flow. The intervention consists of two treatment arms with solutions of identical osmolarity consisting of inhaled salbutamol with Mg sulfate (experimental arm) and with equivalent saline placebo (control arm). The primary outcome is hospitalization to inpatient unit for asthma-related symptoms within 24 h of randomization. *PRAM Pediatric Respiratory Assessment, **Mg magnesium

At the conclusion of the three baseline inhalations, the research nurse assesses eligibility and measures the pre-randomization PRAM score. Eligible children with PRAM ≥5 are approached, and informed consent is obtained. Subjects are randomly allocated to receive three consecutive nebulizations of salbutamol with either diluted Mg sulfate or diluted 5.5 % saline placebo 20 min apart [48] using the Aeroneb™ Go Micropump Nebulizer along with the Idehaler™ Pocket system. Specifically, each treatment utilizes 600 mg (1.2 mL) of Mg sulfate or 1.2 mL 5.5 % saline, 5 mg (1 mL) of salbutamol, and 3.8 mL of sterile water (Fig. 1).

### Randomization and masking

The Research Coordinating Pharmacist at SickKids produced Master Randomization tables, stratified by site and age (≥6 years versus less), using a permuted block randomization of six and eight in a 1:1 ratio of active Mg sulfate to placebo, using random number generating software. The Master Randomization tables are held at the site Research Pharmacies. Consecutively numbered kits are prepared by each pharmacy according to the step-by-step procedure manual provided by Research Coordinating Pharmacist at SickKids. Upon receiving the informed consent, the study nurse obtains the next appropriate numbered study kit from the locked research fridge in the ED (Mg has to be refrigerated) and enters the number in the confidential logbook. Since Mg sulfate solution is hypertonic, 5.5 % saline was chosen as a placebo. Using sterile water as a top-up diluent in both arms produces solutions of identical isotonicity 380 mOsm/L in both study groups. Because the deposition of inhaled drugs is weight-independent [64], a uniform Mg dose is used in all children.

The patients, research nurses and ED physicians are blinded to the treatment assignment. Only the pharmacies are unblinded. Each site is given detailed requirements for drug accountability and handling to ensure compliance with Health Canada regulations. The active Mg and placebo hypertonic saline mixture with salbutamol and sterile water are very similar in volume, color, taste, and smell when nebulized (tested in the research pharmacy at SickKids). To assess blinding, the research nurse and parents are asked at the conclusion of the experimental therapy which intervention they think the child had received.

In case of increasing respiratory distress, IV Mg may be given after the experimental therapy, provided the patient is not hypotensive. In the unlikely event that the patient develops hypotension requiring therapy or apnea and the ED physician feels that the experimental therapy cannot be safely continued, further doses of the experimental treatment are stopped. If these Mg side effects are also accompanied by severe distress and additional IV Mg is warranted, the code may be broken for that patient. Unblinding will only occur if the clinical treatment of the patient will change because the physician knows which arm of the study the patient was previously enrolled. The study PI/local PI and the study nurses will remain blinded. No patients participating in our inhaled Mg study received experimental therapy unblinded.

The study nurses also keep continuous study logs with the characteristics and outcome of the participating and nonparticipating eligible acute asthmatics ≥2 years of age; these logs will also be used to track missed patients, those excluded for criteria, and patients who refused participation, which will help document any selection bias.

### After the intervention

Following the study medication, the patients continue to receive further salbutamol as frequently as clinically warranted as per the treating ED physician. Disposition is also determined by blinded ED physicians. Discharged patients receive a prescription for inhaled salbutamol every 4 h as necessary for the next week and daily oral corticosteroid according to local practice. All participating families receive instructions to visit their primary care provider/ED if salbutamol has to be given more often than every 4 h for increased work of breathing/severe cough and if the respiratory status interferes with usual play/normal speech or activity.

### Outcome measures

#### Primary outcome measure

The primary outcome measure is hospitalization (based on the ED physician’s decision to admit) to an inpatient unit within 24 h of the start of the experimental therapy for persistent respiratory distress or for supplemental oxygen. Children deemed to be admitted but who due to lack of bed availability are never transferred to the ward will be analyzed as hospitalized. Extended ED stays without a decision to admit are not considered admitted.

#### Secondary outcome measures

The two groups will also be compared with respect to the following:

- All cause hospitalization rate by 24 h to examine Mg impact on side effects, such as hypotension necessitating admission.
- Changes in the PRAM, respiratory rate, and oxygen saturation from the start of the first experimental nebulization to 60, 120, 180, and 240 min and the changes in the blood pressure from the first experimental nebulization to 20, 40, 60, 120, 180, and 240 min.
- Number of salbutamol treatments within 240 min of starting study medication.
- An association between hospitalization and age, sex, symptom duration <24 h [60], pre-randomization PRAM score, personal history of atopy, and “acute viral induced wheeze” phenotype [75–81].

#### Other outcomes

Major side effects such as hypotension (systolic blood pressure below the 5th percentile for age) or apnea is tracked as is admission to ICU for airway stabilization.

The clinical outcomes are measured by trained study nurses in the ED. The research nurses also ascertain subsequent return visits/hospitalizations from the telephone follow-ups according to standardized interviewing techniques, as well as from a review of the patient health records at 72 h.

### Sample size

The sample size calculation is based on the targeted minimal significant between-group difference in proportions of hospitalizations of 10 %, which has previously been shown to impact treatment decisions. The estimated probability of hospitalization is based on our fully blinded pilot data (pilot patients form an integral part of this trial), where the overall hospitalization rate is close to 50 %. This is a superiority study in which the adoption of the Mg therapy can only be recommended for future practice if the probability of the primary outcome in the Mg group is significantly lower than in the placebo group. With 408 patients per arm (816 in total), a two-sided test with a type I error of 0.05 will have 80 % power to achieve statistical significance if Mg therapy reduces the probability of hospitalization from 50 to 40 % (that is, absolute reduction of 10 %) [82].

### Analyses

#### Primary analysis

The proportion of patients hospitalized will be compared between treatment groups using a two-sided Fisher’s exact test at the 5 % level. An intention-to-treat analysis will be used with all randomized participants included in the analysis as part of the groups to which they were randomized regardless of whether they completed the study medication or not.

#### Secondary analysis

Secondary analysis will include the following:

- A Fisher’s exact test to compare the rates of all cause admissions in the two arms.
- A repeated measures ANOVA to compare treatment arms with respect to the PRAM score, respiratory rate, heart rate, oxygen saturation, and blood pressure over time. Baseline values will be added to the model as a covariate.
- A Poisson model will be used to compare the number of salbutamol treatments used in the ED in the regression analysis of the two study arms, including interaction terms with the treatment group, which will be used to examine the subgroup effects with respect to the primary outcome. The following variables will be used to define subgroups: age, sex, pre-randomization PRAM score, and personal history of atopy. The statistical tests of hypotheses for the secondary outcomes a) through d) will be two-sided at the 0.00625 level to account for the issue of multiple testing and to maintain an overall type 1 error of 0.05.

#### Interim analysis

To assure safety, one planned interim analysis will be conducted on the first 200 patients randomized (a quarter through the study) conducted by a statistician not involved in the trial and evaluated by the independent data safety monitoring board. The interim analysis will be a one-sided test of the null hypothesis of no difference versus the alternative hypothesis that the probability of hospitalization is higher on Mg therapy at the 0.01 level. That is, we are looking for evidence that Mg therapy is less effective, and the trial will be stopped at an interim analysis only if the null hypothesis is rejected in favor of the control arm. Therefore, the interim analysis is only for safety and not for efficacy, and it will not increase the probability of erroneously rejecting the null hypothesis in favor of Mg therapy at the final analysis. The reason we are doing one-sided (for harm) interim analysis is because if there is early strong evidence that Mg increases the probability of hospital admission, we want to stop the trial. On the other hand, we do not want to stop the trial early for benefit because a smaller sample size will not be convincing.

### Safety

Magnesium blocks the neuromuscular transmission and acts as a CNS depressant. Therefore, the theoretical adverse effects with IV Mg may include a transient drop in blood pressure or apnea [83]. The potential for these problems after nebulized Mg is lower than with IV Mg because this treatment route will result in a lower systemic delivery of Mg (1/4 of the IV dose) and a lower systemic effect. Since hypotension is the only major side effect of IV Mg occurring with appreciable frequency, all patients are monitored using continuous automated blood pressure devices, and if the systolic blood pressure drops below 5^th^ percentile for age, the study will be stopped, treatment given as necessary, and the DSMC will be notified. Of note, a recent Cochrane review of 896 patients given inhaled Mg confirmed the safety of this agent [59]. No child in this study had experienced hypotension or apnea.

All adverse events are being reported to the Hospital for Sick Children Research Ethics Board according to the Hospital for Sick Children’s adverse event reporting requirements. Adverse events will be classified as mild, moderate or severe and as expected or unexpected. Expected adverse events will include cough, respiratory distress, asthma-related hospitalization, IV insertion, sinus tachycardia and bitter/salty taste of the experimental solution. Adverse reactions are managed according to the standard clinical management practices. Furthermore, we plan to document episodes of severe cough necessitating interruption of the experimental therapy for more than approximately 3 min to examine the safety profile of magnesium.

All serious, unexpected adverse drug reactions to the study medication will be reported to Health Canada within 15 calendar days, and those resulting in death or life-threatening events, within 7 calendar days. Serious adverse events include ICU admission, hypotension <5^th^ percentile for age, apnea, and any other medical event which may jeopardize the patients.

Due to the osmolarity of the study solutions being well under 500 mOsm/L throughout nebulization and co-administration of salbutamol, we do not anticipate side effects due to the aforementioned composition of the study solutions. However, should the highly unlikely event of respiratory deterioration occur, the experimental therapy will be discontinued, appropriate additional treatment started, and the event will be reported to the DSMC within 48 h. Salbutamol may cause tachycardia, and this has been the case in many children enrolled to date. However, this has been uniformly well tolerated, and no patient has had to stop/interrupt experimental therapy due to this issue.

The Data Safety and Monitoring Committee (DSMC) consists of a non-study biostatistician, a researcher, and a child health scientist, specifically Dr. Annie Dupuis, Dr. Neil Sweezey, Dr. Patricia Parkin. The members of this committee are not collaborators of this trial. They are notified of all serious adverse events by the study coordinator within 48 h. The DSMC meets once per asthma season or ad hoc if necessary.

### Trial management

The Hospital for Sick Children Research Institute will act as a central repository for all study data and will be responsible for study data management technology and clinical data management services. Dr. Willan will supervise all data analyses. Dr. Schuh takes overall responsibility for the study. The study has a Steering Committee which includes senior research team members: Dr. Nathan Kuppermann (past chair of Pediatric Emergency Children’s Research Network), Dr. Joseph Zorc (senior ED clinician investigator), Dr. Amy C. Plint (past chair of PERC) and Dr. Allan L. Coates (expert in inhaled drug delivery).

## Discussion

If effective, inhaled Mg will represent an effective strategy to minimize morbidity in pediatric refractory acute asthma. Furthermore, the potential for side effects in this study can reasonably be expected to be low, since the inhaled delivery route will result in a lower systemic delivery of magnesium, a lower systemic effect, and a substantially enhanced margin of safety compared to the intravenous administration. To ensure study safety, all severe adverse events will be reported to the Research Ethics Board and the Data Safety and Monitoring Committee as well as to the Steering Committee. A safety interim analysis is planned after the first 200 patients have been randomized and appropriate unblinding procedures have been defined.

Future dissemination of results will include knowledge translation, incorporation into a Cochrane Review, presentation at scientific meetings, and a peer-reviewed publication.

We plan to disseminate the results of this study widely and rapidly to all relevant stakeholders. This will be accomplished via the Pediatric Emergency Research Canada (PERC) network, which has a unique partnership with the Translating Research Emergency Knowledge for Kids (TREKK) group, encompassing 36 general EDs across Canada and 12 PERC sites. In addition, the PERC network is part of the worldwide Pediatric Emergency Research Network (PERN), consisting of 122 hospitals on five continents.

### Trial status

As of 15 September 2015, 207 children had been enrolled.

## Additional file

Additional file 1:
**REB names.** List of REB approving study. (PDF 78 kb)

## Abbreviations

ANOVA: analysis of Variance
ED: Emergency Department
IV: intravenous
Mg: magnesium
PRAM: Pediatric Respiratory Assessment Measure
