# Supplementary material for: Magnesium nebulization utilization in management of pediatric asthma (MagNUM PA) trial: study protocol for a randomized controlled trial
Source: Trials. 2016 May 24;17:261. doi: 10.1186/s13063-015-1151-x (PMC4879727; doi:10.1186/s13063-015-1151-x)
Supplement: Additional file 1: — REB names. List of REB approving study. (PDF 78 kb) [file 13063_2015_1151_MOESM1_ESM.pdf]

REB approval from all of the participating study centres

1. Alberta Children's Hospital Research Institute
   2888 Shaganappi Trail NW, Calgary AB, T3B 6AB, Canada

2. Children's Hospital of Eastern Ontario (CHEO) Research Ethics Board
   401 Smyth Road, Ottawa ON, K1H 8L1, Canada

3. Le Comité d'éthique de la recherche (CÉR) du CHU Sainte-Justine
   3175 chemin de lat Côte-Sainte-Catherine, Montreal QC, H3T 1C5, Canada

4. Stollery Children's Hospital (SCH)
   The Health Research Ethics Board of Alberta (HERBA) –Clinical Trials Committee
   1500-10104 103 Ave NW, Edmonton AB, T5J 4A7, Canada

5. The Children's Hospital of Winnipeg,
   Health Sciences Centre (HSC) Department of Research
   MS7-820 Sherbrook Street, Winnipeg MB, R3A 1R9, Canada.

6. The Hospital for Sick Children Research Ethics Board
   555 University Avenue, Toronto ON, M5G2 L3, Canada

7. The University of British Columbia / Children's and Women's Health Centre of British Columbia Research Ethics Board (UBC C&W REB)
   4480 Oak St, Vancouver BC, V6H 3N1, Canada
